# Renal and Hepatic Functions after A Week of Controlled Ovarian Hyperstimulation during *In Vitro* Fertilization Cycles

**DOI:** 10.22074/ijfs.2016.4689

**Published:** 2016-11-11

**Authors:** Ilaria Romito, Ferdinando Antonio Gulino, Antonio Simone Laganà, Salvatore Giovanni Vitale, Attilio Tuscano, Gianluca Leanza, Giulia Musmeci, Vito Leanza, Agnese Maria Chiara Rapisarda, Marco Antonio Palumbo

**Affiliations:** 1. Department of General Surgery and Medical Surgical Specialties, University of Catania, Catania, Italy; 2. Unit of Gynecology and Obstetrics, Department of Human Pathology in Adulthood and Childhood “G. Barresi”, University of Messina, Messina, Italy; 3. Department of Experimental and Clinical Pharmacology, University of Catania, Catania, Italy

**Keywords:** Infertility, Ovarian Hyperstimulation, Intracytoplasmic Sperm Injection, *In Vitro* Fertilization

## Abstract

**Background:**

One the main aspects of *in vitro* fertilization (IVF) cycle is to avoid any possible systemic damage on women undergoing a controlled ovarian hyperstimulation (COH). The aim of this work is to evaluate renal and hepatic function blood tests in patients undergoing controlled ovarian hyperstimulation during IVF cycles.

**Materials and Methods:**

We performed a prospective cohort analysis. All patients re- ceived a long stimulation protocol with gonadotropin-releasing hormone (GnRH) analogues by daily administration, since the twenty-first day of the previous ovarian cycle followed by COH with recombinant follicle-stimulating hormone (FSH). The daily dose of exogenous gonadotropins for every single patient was modified according to her follicular growth. The oocytes were retrieved during the oocyte pick up and fertilized by standard procedures of intracytoplasmic sperm injection (ICSI). The blood samples to evaluate renal and hepatic functions were taken at the 7^th^ day of ovarian stimulation.

**Results:**

We enrolled 426 women aged between 19 and 44 years, with a mean body mass index (BMI) of 24.68 Kg/m^2^. The mean value of blood urea nitrogen was 14 ± 3.16 mg/ dl, creatinine: 1 ± 0.45 mg/dl, uric acid: 4 ± 1.95 mg/dl, total proteins: 7 ± 3.93 mg/dl, aspartate aminotransferase: 18 ± 6.29 mU/ml, alanine aminotransferase: 19 ± 10.41 mU/ ml, alkaline phosphatase: 81 ± 45.25 mU/ml, total bilirubin 1 ± 0.35 mg/dL. All of the results were considered as a normal range following the Medical Council of Canada.

**Conclusion:**

Our data suggest that, unlike ovarian hyperstimulation syndrome (OHSS), COH patients did not show any alteration to renal and hepatic functions.

## Introduction

The correlation between renal and hepatic damages and ovarian stimulation is not well understood, and so far data about ovarian hyperstimulation syndrome (OHSS) are not enough robust. As widely evidenced, OHSS is a rare iatrogenic complication of ovarian stimulation, that usually happens during an *in vitro* fertilization (IVF) cycle, luteal phase or early pregnancy. OHSS has been known since 1943, when recombinant gonadotrophins recombinant follicle-stimulating hormone (rFSH), and recombinant luteinizing hormone, (rLH) were used for the first time to induce ovulation (1, 2). OHSS generally occurs only after exposure to human chorionic gonadotropin (hCG) and its mortality rate is between 1 in 45.000 and 1 in 500.000 (3), and it has a morbidity even higher though not accurately quantified. Based on the clinical presentation, laboratory and ultrasound findings, OHSS is classified into three categories (mild, moderate and severe) and five grades (1 ± 5) of severity (4). The initial symptoms are abdominal bloating and pain; the ovarian size usually is <8 cm. In severe clinical presentations, the patients suffer from ascites, oliguria and haemoconcentration; in these cases, ovarian size is usually >12 cm. The mild form has rather high incidence considering that it affects up to 33% of woman undergoing to IVF cycles, while the moderate or severe OHSS complicates 3-8% of IVF cycles (5). Altered liver function tests have been considered to be a rare expression of the severe form of OHSS, because it may induce microvascular thrombosis and liver tissue ischemia leading to hepatic dysfunction (6).

An accurate evaluation of the patient before an IVF technique includes an hysteroscopy for diagnostic as well as therapeutic purpose (7-9), but also the study of any thrombophilic genetic nucleotide polymorphisms (10); it is mandatory to study male partner, evaluating accurately semen parameters (11).

The main risk of a stimulation protocol for an IVF cycle could be considered OHSS, so to date it is recommended to accurately check renal and hepatic functions through blood tests, in order to suspect this condition as early as possible. Considering also that most of the women try repeated cycles of IVF, due to the common failure of the technique, it is important to know whether the normal stimulation protocol (which does not hesitate into OHSS) could determine renal and/or hepatic damages. To the best of our knowledge, no study has yet investigated this point, since most of the available data were focused on OHSS. In the light of this evidence, we think that it is extremely important to know if even normal stimulation protocols with controlled ovarian hyperstimulation (COH) may lead to renal and/or hepatic altered functions, also taking into account the “basal risk” before the start of another IVF cycle. Thus, the aim of this work is to evaluate renal and hepatic function blood tests in patients undergoing COH during IVF cycles.

## Materials and Methods

We performed this single-center prospective cohort study at the Department of General Surgery and Medical Surgical Specialties of the University of Catania (Italy), between July 2012 and August 2015. We enrolled women IVF for primary or secondary infertility, considering the development of OHSS as exclusion criteria. Each patient was informed about the procedures and signed an informed consent allowing data collection for research purposes.

All patients received a long stimulation protocol, which involved the pituitary desensitization with gonadotropin-releasing hormone (GnRH) analogues by daily administration since the twenty-first day of the previous ovarian cycle. When we reached pituitary desensitization, indicated by serum estradiol (E_2_) level <40 pg/ml and follicular diameter <10 mm, we started ovarian stimulation with rFSH (GONAL-f, Merck Serono Europe, UK). We modified the daily dose of exogenous gonadotropins for every single patient according to her follicular growth.

We administrated 10000 UI of hCG (Gonasi HP, IBSA Farmaceutici Italia, Italy) when the two largest follicles reached a minimum diameter of 16 mm with a peak of E_2_ of about 1600 pg/ml. We performed oocyte pick-up about after 36 hours by transvaginal ultrasound-guided needle aspiration of the follicles.

The oocytes were fertilized by standard procedures of intracytoplasmic sperm injection (ICSI). After 72 hours embryos were transferred into the uterus. All patients underwent luteal phase support with progesterone and low-dose of acetylsalicylic acid. The pregnacy test was performed after 12 days. 

We evaluated renal and hepatic function blood tests (creatinine, blood urea nitrogen, total protein, uric acid, transaminases, total bilirubin, alkaline phosphatase) at the day 7^th^, since ovarian stimulation with rFSH.

The study was designed in accordance with the Helsinki Declaration, confirming the Committee on Publication Ethics (COPE) guidelines and it was approved by the Institutional Review Board (IRB) of the university hospital, where this work was performed. All designs, analyses, interpretation of data, drafting and revisions followed the Strengthening the Reporting of Observational Studies in Epidemiology (STROBE) Statement: guidelines for reporting observational studies, available through the EQUATOR (Enhancing the QUAlity and Transparency Of health Research) network. All the results were considered as normal range following the standard values defined by Medical Council of Canada (12).

## Results

We recruited 426 women aged between 19 and 44 years (mean age: 32.88 years) that met inclusion and exclusion criteria. We excluded from the study four patients that have developed OHSS during IVF procedures. Mean body mass index (BMI) of the cohort was 24.68 Kg/m^2^ . As reported in Table 1, we measured aspartate aminotrasferase (AST) in 393 patients (93.1%), alanine aminotrasferase (ALT) in 393 patients (93.1%), alkaline phosphatase in 402 patients (95.3%), total bilirubin in 400 patients (94.8%), blood urea nitrogen in 392 patients (92.9%), creatinine in 400 patients (94.8%), uric acid in 350 patients (82.9%), total proteins in 390 patients (92.4%). 

**Table 1:** Renal and hepatic blood tests. Standard values refer to Medical Council of Canada, Appendix C - Clinical Laboratory Tests (2010)


Variables	Mean ± SD	Standard values

Blood urea nitrogen (mg/dL)	14.26 ± 3.16	7.0-22.0
Creatinine(mg/dL)	0.78 ± 0.45	0.57-1.02
Uric acid(mg/dL)	3.77 ± 1.95	3.0-7.0
Total protein(mg/dL)	7.41 ± 3.93	6.7-8.7
Aspartate aminotrasferase(mU/mL)	18.37 ± 6.29	18-40
Alanine aminotrasferase(mU/mL)	19.06 ± 10.41	17-63
Phosphatase alkaline (mU/mL)	81.49 ± 45.25	38-126
Total bilirubin(mg/dL)	0.64 ± 0.35	<1.5


## Discussion

Pregnancy is a physiological condition that may predispose itself to abnormal renal and hepatic functions (13). During pregnancy, the increased blood flow in the liver should reduce transaminases (14), but there are several pathologic conditions such as chronic intrahepatic cholestasis and HELLP (Hemolysisis, Elevated Liver enzyme levels and Low Platelet) syndrome in which there is an increase in AST and ALT (15, 16). Although several studies already investigated renal and hepatic alterations in women who developed OHSS, to the best of our knowledge this is the first report of liver and kidney function during COH. As previously evidenced by Kopylov et al. (17), IVF pregnancies had more elevated AST values compared to spontaneous ones. Generally, liver blood tests are elevated in about 3-5% of all pregnancies (18). Giugliano et al. (19) reported a case of liver failure after four cycles of COH and subsequent intrauterine insemination (IUI): in this case, the patients developed severe HELLP syndrome during the third trimester, allowing authors to hypothesize a correlation between COH and liver failure. Considering HELLP syndrome, hepatic damage could be due (at least in part) by endothelial dysfunction, since it was already demonstrated that increased angiotensin and pro-inflammatory cytokines may lead to hepatic ischemia (20). Furthermore, as documented by Obrzut et al. (21), liver damage severity could be foretold by high value of estradiol during ovarian stimulation, in direct proportion with risk of developing OHSS. Based on these data, we could hypothesize a link between high estradiol values during ovarian hyperstimulation and histopathological liver changes in woman with severe OHSS, similar to those already determined during oral contraceptive therapy (22-24).

Recent data (25, 26) demonstrated that the trends in liver and renal function tests could be affected by single- or double-dose methotrexate in case of ectopic pregnancy after fresh IVF embryo transfer cycles; for this reason, liver and renal function tests have to be carefully evaluated in these patients.

Another specific condition is represented by cases of IVF treatment in renal transplanted patients (who have already altered renal function) and nephrotic syndrome: these patients have higher risk to develop OHSS (27), so they have to be monitored strictly to avoid it.

## Conclusion

The ovarian hyperstimulation, even if controlled, determines physiological modifications and could lead to hemodynamic changes in kidney and liver. Based on that, in our study we expected changes on the parameters of liver and kidney function in women undergoing COH. Our data demonstrated that no patient developed hepatic and/or renal damage, suggesting a good safety profile for COH. Nevertheless, several limitations of this study should be taken into account: first of all, we did not measure all parameters in every patients, although rate of evaluation was above 90% in 7/8 of them; second, we modified the daily dose of exogenous gonadotropins for every single patient according to her follicular growth; finally, our cohort included patients with different ages (between 19 and 44 years) and different types of infertility (primary or secondary). For these reasons, it is required to further study on larger cohorts, with greater statistical power, to accurately clarify the risk of hepatic and/or renal damage during COH.
